# BEYOND THE CLASSROOM: A STUDENT INTERNSHIP MODEL THAT BUILDS AGE FRIENDLINESS AND CAREER READINESS

**DOI:** 10.1093/geroni/igac059.449

**Published:** 2022-12-20

**Authors:** Skye Leedahl, Kristin Fratoni Souza, Alexandra Morelli

**Affiliations:** University of Rhode Island, Kingston, Rhode Island, United States; University of Rhode Island, Kingston, Rhode Island, United States; University of Rhode Island, Kingston, Rhode Island, United States

## Abstract

For seven years, the University of Rhode Island (URI) Engaging Generations Cyber-Seniors Program has utilized university student mentors through an internship format and integration within service learning courses. In 2019, community interest in the program significantly increased as did student interest in gerontology internships. This led us to develop a robust internship program focused on building age and digital inclusivity across campus and the state of RI. The program integrates a three-pronged approach where students complete field hours within the URI Cyber-Seniors program, focus on enhancing the Career Readiness Competencies employers seek in graduates today as determined by the National Association of Colleges & Employers, and complete the components necessary to earn the Geriatric Education Center Interprofessional Teamwork in Geriatrics and Gerontology Certificate. This process has enabled 350 students from different majors, including Pharmacy and Human Development & Family Science, to complete internship experiences ranging from 30 to 210 hours.

